# Autologous oocyte cryopreservation in women aged 40 and older using minimal stimulation IVF

**DOI:** 10.1186/s12958-015-0110-4

**Published:** 2015-10-06

**Authors:** John J. Zhang, Simon Choo, Mingxue Yang

**Affiliations:** Reproductive Endocrinology and Infertility, New Hope Fertility Center, 4 Columbus Avenue, New York, NY USA

**Keywords:** IVF, Oocyte cryopreservation, Minimal ovarian stimulation, Mild ovarian stimulation, Oocyte freezing

## Abstract

**Background:**

The value of oocyte cryopreservation in older women remains controversial. The aim of this study was to report the oocyte freezing experience in women aged 40 and older at a single fertility center.

**Findings:**

One hundred fifty eight women (mean age 43.9 ± 0.2) who underwent minimal ovarian stimulation IVF were enrolled. IVF protocol included the use of clomiphene citrate (50 mg/day) or letrozole (2.5 mg/day) with or without low dose gonadotropins (started at 75 IU/day and increased as needed to 150 IU/day). 584 retrieved oocytes (2.1 ± 0.15 per patient) yielded 532 mature MII oocytes that were frozen. After thawing and fertilization by ICSI, a total of 344 embryos (1.9 ± 0.1 per patient) were formed. A total of 57 relatively good embryos were transferred and yielded three live births (5.3 % per embryo transfer), three spontaneous abortions, and one chemical pregnancy.

**Conclusions:**

These data are important in counseling older women who desire autologous oocyte freezing.

## Introduction

Oocyte cryopreservation has wide clinical implications especially for women who have no partner or stand to lose ovarian function due to aging [1]. Following embryo cryopreservation, oocyte cryopreservation is the second most commonly used method of fertility preservation for medical indications [1]. In January 2013, the American Society for Reproductive Medicine declared that the technique of oocyte cryopreservation (egg freezing) is no longer experimental [2]. In young women, there is good evidence that fertilization and pregnancy rates are similar to IVF/ICSI with fresh oocytes when vitrified/warmed oocytes are used as part of IVF/ICSI. Although data are limited, no increase in chromosomal abnormalities, birth defects, and developmental deficits has been reported in the offspring born from cryopreserved oocytes when compared to pregnancies from conventional IVF/ICSI and the general population.

Over the past decade, vitrification has been developed as an alternative to slow-freeze [3, 4]. Most data in women suggest that post-thaw survival rates of vitrified oocytes are much higher than oocytes that underwent slow-freeze [3, 4]. Vitrification protocols use high initial concentrations of cryoprotectant and ultra-rapid cooling to solidify the cell into a glass-like state in order to prevent the formation of ice crystals. Vitrification is currently being applied to the cryopreservation of embryos, oocytes, and ovarian tissue [2, 5]. Embryo cryopreservation and oocyte cryopreservation in young women are well-established techniques among IVF centers [6]. However, data pertaining to autologous oocyte cryopreservation by vitrification in older women, especially those older than 40, remain scarce.

Minimal and mild ovarian stimulation IVF usually refers to the use of low-dose gonadotropins with or without a sequential administration of clomiphene citrate or letrozole [7]. Typically, minimal/mild ovarian stimulation usually refers to gentle stimulation protocols that yield a maximum of five to six oocytes [7]. It has been suggested that the relatively small number of oocytes obtained after gentle ovarian stimulation may represent the best of the cohort in a given cycle [8]. However, data pertaining to the outcome of autologous oocyte freezing, in particular live birth, in older women using minimal/mild ovarian stimulation IVF is understudied. Thus, the purpose of this manuscript was to report the outcome of frozen oocytes by vitrification in women aged 40 or older who underwent minimal/mild stimulation IVF.

### Materials and methods

This is a retrospective chart review study that involved 158 women aged 40 or older who underwent minimal/mild ovarian stimulation IVF at a single fertility center (New Hope Fertility Center, New York). Reasons for autologous oocyte cryopreservation included: women who had no male partner, women who desired to preserve their future ability to have children because they wanted to delay childbearing, women who had fear of losing all oocytes due to aging, women with a family history of early menopause fearing that their oocytes would be depleted at an early age. All patients were counseled that the efficacy and the success (i.e., achieving a live birth) of autologous oocyte freezing is understudied in women aged over 40. They were also informed that the probability of getting pregnant with frozen oocyte could be low after the age of 40 although specific percentages could not be quoted. The studay was approved by the Institutional Review Board of New York Downtown Hospital (IRB approval reference number: JZ-09-08) [9].

After oral contraceptive pill pre-treatment for approximately 3 weeks and adequate suppression, minimal/mild ovarian stimulation was started with an extended regimen (from cycle day 3 until the day before triggering) of clomiphene citrate (50 mg/day orally) or letrozole (2.5 mg/day) in conjunction with low dose of gonadotropin injections (75 IU/day and increased as needed to 150 IU/day) (Bravelle and/or Menopur, Ferring, Parsippany, NJ; Follistim, Merck, White House Station, NJ; or Gonal F, EMD Serono, Rockland, MA) starting on cycle day 4 to 7 depending on the response. Ovarian response was monitored using serial transvaginal ultrasound and serum blood measurement for estradiol, progesterone and luteinizing hormone. No hypothalamic-pituitary ovulation suppression using GnRH agonist or antagonist was used in this protocol. The final maturation of oocytes was induced by a nasal GnRH agonist (Synarel nasal spray 2 mg/mL, Pfizer, New York, NY) when the lead follicle is >18 mm. Retrieved oocytes were checked for maturity and mature oocytes were cryopreserved by vitrification.

Oocytes were vitrified using the Kitazato commercial kit (Dibimed Biomedical Supply S.L., Valencia, Spain) as previously descibed [10–12]. The oocytes were aspirated with a minimal volume of HEPES culture media then placed in a micro-droplet containing the Basic Solution. The oocytes were allowed to acclimate to the new solution for approximately 10–30 s then placed in a drops of Equilibration Solution containing 7.5 % (vol/vol) ethylene glycol (EG) + 7.5 % dimethyl sulfoxide (DMSO) at room temperature for 15 min. After 6 min, the oocytes were transferred into a Vitrification Solution drop containing 15 % EG + 15 % DMSO + 0.5 M sucrose for a total of 50 s before being loaded into a Cryotop (fine and thin strip) that is quickly plunged into liquid nitrogen.

Oocyte thawing and fertilization by ICSI was performed for all couple when using either the male partner or donor sperm. While all semen parameters are important during an ICSI cycle, motility is the most important because it is an indication of living sperm. For this study all sperm injected into oocytes were motile. When choosing motile sperm for injection, basic head, neck and tail morphology were considered, and in all cases the best sperm available were chosen over any dysmorphic sperm in the samples. The embryos were cultured in a single global total medium (LifeGlobal Group LLC) that contains proteins, salts, amino acids, buffer (NaHCO3), glucose, pyruvate, lactate and antibiotics (gentamicin). All embryos were placed in incubators containing 5 % CO2 that results in a physiologic pH of approximately 7.30.

A single embryo was transferred in a subsequent artificially prepared cycle with oral estradiol (Estrace, Actavis Pharma, Inc, Parsippany, NJ) [13]. All patients started oral estradiol at 4 mg/d orally for 10 days starting on the third day of menstruation. The dose of Estrace was subsequently increased to 6–8 mg/d in cases where serum estradiol level was less than 200 pg/mL and/or when ultrasound images showed an endometrial thickness of less than 7 mm. Oral estradiol treatment was then continued for 7–14 days until the endometrial thickness was more than 7 mm. Progesterone vaginal insert (prometrium) 200 mg three time daily was administered to support the luteal phase and oral estradiol was continued along with progesterone. Embryo transfer was performed on the 5th day of progesterone supplementation for cleavage stage embryos and on the 7th day of progesterone supplementation for blastocyst embryos. All embryo transfers were performed under in ultrasound guidance to ensure correct placement, which is 1–2 cm from the uterine fundus.

Data were presented as mean ± standard error of the mean. The main outcome was live birth per embryo transfer. Pearson correlation was performed to evaluate the association between age and clinical parameters such as day 3 FSH, peak serum estradiol level, number of oocytes retrieved, and number of mature metaphase II oocytes. Pearson correlation was also performed to evaluate the association between BMI and the number of oocytes retrieved and mature metaphase II oocytes. Statistical significance was declared if the two-sided *p*-value was ≤ 0.05. Statistical analyses were performed using statistical software GraphPad Prism six (GraphPad Software, Inc. La Jolla, CA, USA).

## Results

The participants had a mean age of 43.9 ± 0.2 years (range: 40 to 49 years), mean body mass index of 23.6 ± 0.4 kg/m^2^ (range: 17 to 39 kg/m^2^) and a mean baseline day 3 FSH of 12.6 ± 0.6 mIU/mL (range: 2 to 48 mIU/mL). The mean number of days of ovarian stimulation with gonadotropins injections was 6.1 ± 0.3 and the mean peak estradiol level 660.7 ± 37.4 pg/mL.

**Fig. 1:**
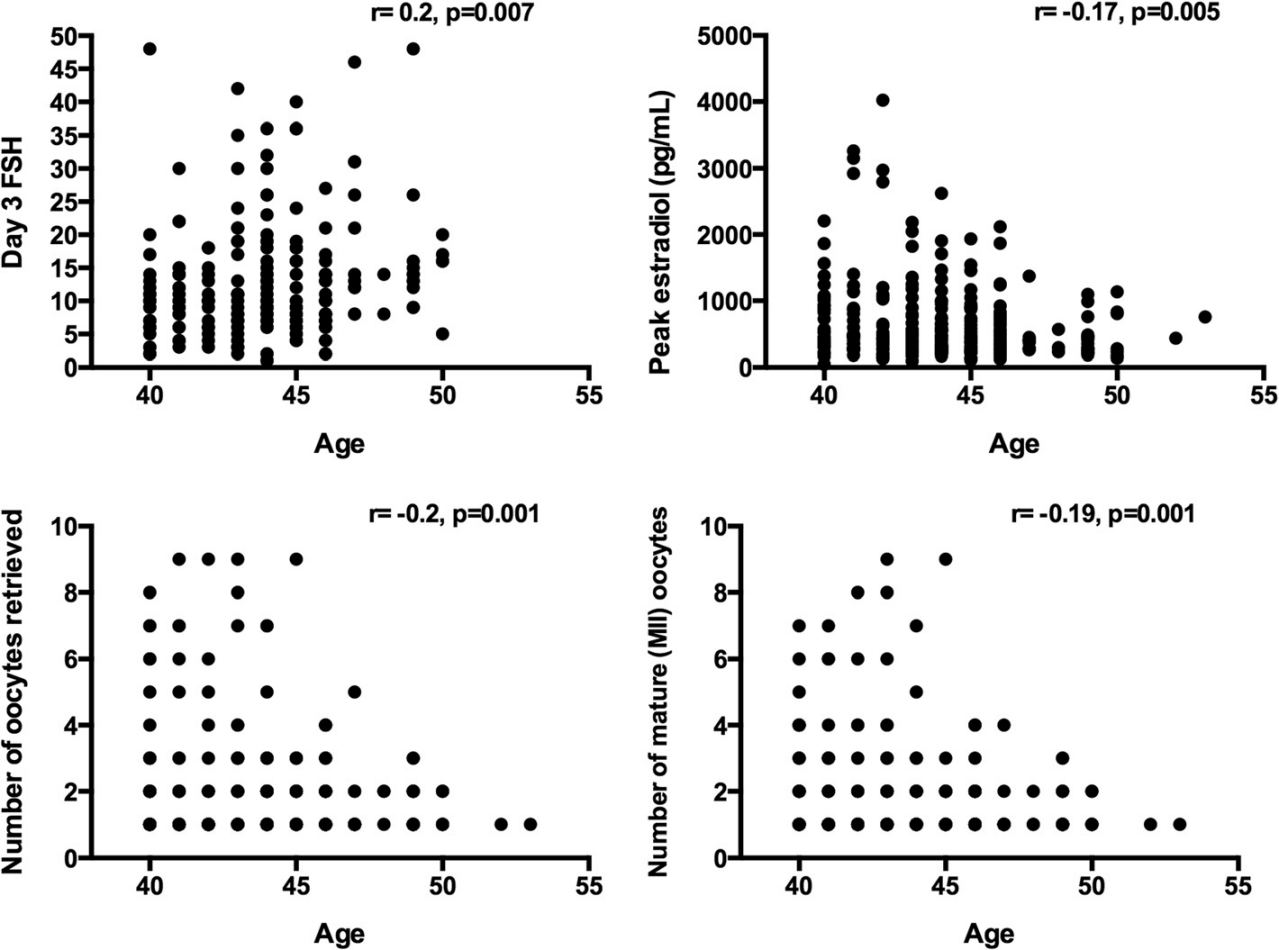
Correlations between age and day 3 FSH, peak estradiol levels, number of oocytes retrieved and number of mature metaphase II (MII) oocytes. There was a positive correlation between age and day 3 FSH; however, there was a negative correlation between age and 1) peak estradiol level, 2) the number of oocytes retrieved, and 3) the number of mature MII oocytes

There was a positive correlation between age and day 3 FSH (*r* = 0.2, *p* = 0.007). There was also a negative correlation between age and peak estradiol level (*r* = −0.17, *p* = 0.005), the number of oocytes retrieved (*r* = −0.2, *p* = 0.001), and the number of mature metaphase II oocytes (*r* = −0.19, *p* = 0.001) (Fig. 1). There was no correlation between BMI and the number of oocytes retrieved (*r* = −0.05, *p* = 0.6) or the number of mature metaphase II oocytes (*r* = −0.05, *p* = 0.6).

A total of 584 oocytes (2.1 ± 0.15 per patient) were retrieved; 532 of which were mature metaphase II oocytes that were cryopreserved by vitrification. Four patients had no (i.e., zero) oocytes retrieved. After thawing and fertilization of the 532 mature oocytes by ICSI, 485 embryos (1.9 ± 0.1 per patient) were formed. Table 1 reports the different stages of embryos formed and the number of embryos formed in each category. A total of 57 relatively good embryos were transferred and yielded six clinical pregnancies (10.5 % clinical pregnancy rate per transfer) and one chemical pregnancy. Of the clinical pregnancies, three pregnancies yielded live births (5.3 % live birth rate per transfer) and three spontaneous abortions.

**Table 1:** Number of embryos in each stage of development following oocyte thaw and fertilization by ICSI in women aged 40 and older who underwent minimal/mild ovarian stimulation IVF

Embryo stage	Numberof embryos
Completely hatched blastocyst	2
Hatched blastocyst	17
Expanded blastocyst	11
Blastocyst	17
Early blastocyst	1
Cavitating blastocyst	14
Morula	9
Compacted morula	25
14-cell embryo	1
10-cell embryo	1
9-cell embryo	3
8-cell embryo	58
7-cell embryo	13
6-cell embryo	34
5-cell embryo	19
4-cell embryo	48
3-cell embryo	15
2-cell embryo	32
1-cell embryo	18
1 pronuclei	10
2 pronuclei	6
3 pronuclei	17
4 pronuclei	9
Multi-pronuclei	2
Degenerated	103

## Discussion

The number of women over 40 years of age seeking infertility treatment has been steadily increasing in the past decade due to postponing childbearing in career women as well as the desire of pregnancy in couples starting a second family [14]. The decision to delay childbearing has biologic consequences. While the onset and progression of reproductive decline is unique for each individual, it is inevitable once a woman reaches her 30’s years. Women with diminished ovarian reserve have decreased response to gonadotropins, require higher doses of gonadotropins, have higher miscarriage rates, and have lower pregnancy/live-birth rates [15–18]. For those who desire to delay childbearing and have good ovarian reserve, oocyte freezing represents a good option [2]. However, while the success of oocyte freezing may be as high as 45 % [19], it declines rapidly once a woman reaches age 35 [15].

Several studies have evaluated the impact of age on the outcome of oocyte cryopreservation [20] and demonstrated that success rates with oocyte cryopreservation via either slow-freeze or vitrification decline with maternal age [20–22]. A study (*n* = 450 patients) on women who underwent oocyte thaw cycles using previously vitrified supernumerary oocytes found that maternal age was inversely correlated with delivery rates [22]. Another study (*n* = 182 participants) reported that ongoing pregnancy rate using oocyte vitrification/warming cycles was negatively correlated with age [19]. In that study, a significant difference in cumulative pregnancy rate was found between age group < 34 years and age group 41–43 years (*p* = 0.006).

Several studies using slow-freeze protocols suggested that success rates were lower with advanced maternal age. In a large cohort study (*n* = 2046 patients), oocyte survival was similar in women < or > than 38 years old [20]. As expected, women aged > 38 had significantly lower implantation rates (6.5 % vs. 10.9 %, *p* = 0.01) and pregnancy rates (10.1 vs. 18.7 %, *p* = 0.02) compared to women aged < 38 [20]. Unlike our study, the mean patient age at retrieval was 35.04 ± 3.97 years (range, 21–45 years). Another study (*n* = 342 patients) using a slow-freeze protocol reported pregnancy rates of oocyte cryopreservation in three groups of women by age: ≤34 years (group A, mean age 31.1 ± 2.50), 35–38 years (group B, mean age 36.4 ± 1.07), and ≥ 39 years (group C, mean age 40.6 ± 1.36) [21]. In groups A, B and C respectively, the implantation rates were 16.7, 11.6, and 10.8 %, the pregnancy rates per thaw cycle were 24.3, 18.9, and 16.1 %, and the pregnancy rates per embryo transfer were 27.7, 21.4, and 17.6 %. The outcomes were comparable among the three groups although there was a trend for all outcomes to be lower in older women (*p* > 0.05 for all) [21].

With the efficiency of vitrification, autologous oocyte banking appears to be a solution for later motherhood. This report presents a description on the quality and outcome of embryos following oocyte cryopreservation in women aged 40 and older (age range: 40–52) who underwent gentle ovarian stimulation IVF at a single fertility center. It also reports the pregnancy rate per single embryo transfer of those who thawed and fertilized their oocytes. Although these data are useful in counseling older women who desire autologous oocyte freezing, a lingering question for patients and clinicians is: What is the upper age limit to offer oocyte cryopreservation? Because one cannot place an absolute value on childbearing, the upper age limit for considering oocyte cryopreservation may vary according to individual preferences, values, and finances. Given the data presented in the current study, it is reasonable to counsel women aged more 40 that, if she forms embryos by minimal stimulation IVF, each embryo transferred has a very low chance (only 5.3 %) of yielding a live birth. In conclusion, this report extends the body of literature reported on oocyte cryopreservation in younger women and in women who undergo conventional IVF to those undergoing minimal stimulation IVF.
